# Mouth Washing Impaired SARS-CoV-2 Detection in Saliva

**DOI:** 10.3390/diagnostics11081509

**Published:** 2021-08-22

**Authors:** Monique Melo Costa, Nicolas Benoit, Hervé Tissot-Dupont, Matthieu Million, Bruno Pradines, Samuel Granjeaud, Lionel Almeras

**Affiliations:** 1 Unité de Parasitologie et Entomologie, Département de Microbiologie et Maladies Infectieuses, Institut de Recherche Biomédicale des Armées, 13005 Marseille, France; mcosta.monique@gmail.com (M.M.C.); nicobenoit73@hotmail.com (N.B.); bruno.pradines@gmail.com (B.P.); 2 Aix Marseille Univ, IRD, SSA, AP-HM, VITROME, 13005 Marseille, France; 3 IHU Méditerranée Infection, 13005 Marseille, France; herve.tissot-dupont@ap-hm.fr (H.T.-D.); Matthieu.MILLION@ap-hm.fr (M.M.); 4 Centre National de Référence du Paludisme, 13005 Marseille, France; 5 Aix-Marseille-Université, IRD, MEPHI, IHU Méditerranée Infection, 13005 Marseille, France; 6 CRCM Integrative Bioinformatics Platform, Centre de Recherche en Cancérologie de Marseille, INSERM, U1068, Institut Paoli-Calmettes, CNRS, UMR7258, Aix-Marseille Université UM 105, 13009 Marseille, France; samuel.granjeaud@inserm.fr

**Keywords:** saliva, COVID-19 diagnosis, coronavirus, SARS-CoV-2

## Abstract

Background: A previous study demonstrated the performance of the Salivette^®^ (SARSTEDT, Numbrecht, Germany) as a homogeneous saliva collection system to diagnose COVID-19 by RT-qPCR, notably for symptomatic and asymptomatic patients. However, for convalescent patients, the corroboration of molecular detection of SARS-CoV-2 in paired nasopharyngeal swabs (NPS) and saliva samples was unsatisfactory. Objectives: The aim of the present work was to assess the concordance level of SARS-CoV-2 detection between paired sampling of NPSs and saliva collected with Salivette^®^ at two time points, with ten days of interval. Results: A total of 319 paired samples from 145 outpatients (OP) and 51 healthcare workers (HW) were collected. Unfortunately, at day ten, 73 individuals were lost to follow-up, explaining some kinetic missing data. Due to significant waiting rates at hospitals, most of the patients ate and/or drank while waiting for their turn. Consequently, mouth washing was systematically proposed prior to saliva collection. None of the HW were diagnosed as SARS-CoV-2 positive using NPS or saliva specimens at both time points (*n* = 95) by RT-qPCR. The virus was detected in 56.3% (*n* = 126/224) of the NPS samples from OP, but solely 26.8% (*n* = 60/224) of the paired saliva specimens. The detection of the internal cellular control, the human RNase P, in more than 98% of the saliva samples, underlined that the low sensitivity of saliva specimens (45.2%) for SARS-CoV-2 detection was not attributed to an improper saliva sample storing or RNA extraction. Conclusions: This work revealed that mouth washing decreased viral load of buccal cavity conducting to impairment of SARS-CoV-2 detection. Viral loads in saliva neo-produced appeared insufficient for molecular detection of SARS-CoV-2. At the time when saliva tests could be a rapid, simple and non-invasive strategy to assess large scale schoolchildren in France, the determination of the performance of saliva collection becomes imperative to standardize procedures.

## 1. Introduction

The emergence in December 2019 of severe acute respiratory syndrome coronavirus 2 (SARS-CoV-2), responsible for coronavirus disease 2019 (COVID-19), in Wuhan, China, and its spread all over the world, raised an urgent need for developing diagnostic tests to detect and to isolate positive cases. The nasopharyngeal swabs (NPSs) were quickly established as the reference method for sample collection of COVID-19 diagnosis based on RT-qPCR tests [1]. However, NPS collection causes discomfort to patients and is contraindicated in particular cases, including blood clotting diseases or deviated septum [2]. Thereby, it is less and less well accepted by the population [3]. Additionally, NPS sampling, which requires specialized consumables and trained medical personnel, exposes these professionals to risk of virus infection [3]. The cumulative drawbacks of NPS sampling conducted to propose alternative biological samples for SARS-CoV-2 screening [4]. Among the different sources of sample collection tested, the saliva was the best accepted specimen by patients, notably for repeat testing [5,6]. This painless, non-invasive and simple self-collection method could became a suitable alternative for SARS-CoV-2 screening tests [7]. Although pioneering studies comparing the performance of RT-qPCR detection of SARS-CoV-2 between NPS and saliva samples obtained mitigate concordances [8,9], more recent works tended to conclude the relevance of using saliva for COVID-19 diagnosis [6,10]. Different methods and tools have been assessed for saliva collection, from direct drooling in plastic tubes [11,12] until the use of dedicate devices [13,14]. The diversity of the saliva sampling system used could explain, in part, the heterogeneity of COVID-19 diagnosis performances.

To facilitate result comparisons, a standardization of saliva collection is required. In a previous study, we demonstrated the performance of a new saliva collection system, consisting in roll cotton and called Salivette^®^ (Neutral Salivettes^®^, SARSTEDT, Numbrecht, Germany) as a homogeneous saliva collection system to diagnose COVID-19 by RT-qPCR [15]. The same protocol applied for SARS-CoV-2 diagnosis in a previous study [15] revealed a significantly higher sensitivity for SARS-CoV-2 detection in saliva collected with Salivettes compared to NPS. The only difference between this previous study and the present one was the realization of mouth washing prior to saliva collection. The principle of this device consists of the use of a roll cotton, which is introduced in the patient mouth for a few minutes to soak it with saliva. The saliva is then retrieved after a quick centrifugation. This rapid, easy to use self-collection device appears well adapted for mass-testing. Salivette^®^ presents the advantage to be a hygienic device preventing saliva droplets or dripping off the collection tube, and the saliva retrieved is not viscous, facilitating pipetting.

Here, we assessed the concordance level of SARS-CoV-2 detection between paired sampling of NPSs and saliva collected with Salivette^®^ at two time points, with ten days of interval. Sampling collection including outpatients (OP, *n* = 145) and healthcare workers (HW, *n* = 51), started at the end of July 2020. This summer period corresponded to the second wave initiation of COVID-19 outbreak in the south of France. The sudden increase in subject led to significant waiting time at hospital. Thereby, most of the patients ate and/or drank in waiting for their turn, involving that mouth washing was systematically proposed to all individuals enrolled in the study prior to saliva sampling. The consequences of mouth washing on SARS-CoV-2 detection in saliva were assessed.

## 2. Materials and Methods

### 2.1. Ethical Statement

The study protocol was reviewed and approved by the Ile de France 1 ethical committee (N°2020-A01249-30 protocol, 6 August 2020). Demographics, clinical data and samples were collected uniquely after the understanding of the study protocol and consent acknowledgement by the participants. A case report form, including health status and clinical data of each participant was provided. All participant information and samples were anonymized prior their use. The sample manipulations were carried out under class II biological safety cabinets MSC-Advantage^TM^ (Thermo Fischer Scientific, Villebon sur Yvette, France).

### 2.2. Individual Recruitment

*Outpatients* (*OP*). During the period from 23 July 2020 to 21 September 2020, outpatients consulting to the Institut Hospitalo-Universitaire (IHU) Méditerranée Infection (Marseille, France), and diagnosed positive for SARS-CoV-2 by nasopharyngeal swabs (NPSs) in the last 5 days were invited to enroll in the research study. Saliva collections were carried out at the day of patient inclusion (D0) and ten (D10) days later. *Healthcare workers* (*HW*). Healthcare workers without fever or respiratory symptoms were invited to enroll in the study. Saliva collection was carried out at the day of individual inclusion (D0) and ten (D10) days later. A NPS was performed to all participants from OP and HW groups, to determine to their COVID-19 status the same day of saliva collections. Individuals under 18 years old, non-French speaking, pregnant women and individuals suffering of Gougerot-Sjögren Syndrome, a systemic autoimmune disease characterized by damage to salivary glands, were excluded.

### 2.3. NPS Management

A standard protocol was applied for NPSs collection using nasal swabs with viral transport medium (Pacific Laboratory Products, Blackburn, Australia), as previously described [16].

### 2.4. Saliva Collection

A bottle of spring water (Cristaline, Cairanne, France) was given to each participant who performed a quick mouthwash to eliminate drink and food remains prior to saliva sampling. Saliva was collected using Salivette^®^ under the supervision of a medical biology laboratory technician. The cotton roll was directly introduced in the mouth without handling and then kept 2 min in the mouth’s participant who soaked the cotton by doing circular movements, prior to replacing it into the stopper part of the Salivette^®^ tube. The samples were refrigerated on ice at the collection site and stored in these conditions until they arrived in the laboratory. The samples were divided into aliquots and stored at −80 °C until RNA extraction and subsequent PCR analysis. The sample processing time never exceeded 6 h. 

### 2.5. Saliva Sample Preparation

Salivette^®^ cotton rolls were prepared as previously described [15]. If the retrieved saliva volume, after centrifugation, was less than 150 µL, 500 µL of ultra-pure water were loaded at the top of the cotton roll and the Salivette^®^ was then once again centrifuged at 1500× *g* for 2 min at 4 °C. The addition of ultra-pure water was done to 25 saliva samples.

### 2.6. RNA Extraction

Viral RNA was extracted from 150 µL of the samples (NPS fluids or saliva) using NucleoMag^®^ Pathogen Isolation kit (Macherey-Nagel GmbH & Co, Düren, Germany). The nucleic acid extraction was fully automated using KingFisher™ Flex system (ThermoFisher Scientific, Villebon Courtaboeuf, France), within 28 min, according to the manufacturer’s instructions. The RNA was recovered in 75 μL of elution buffer and used directly as a template in RT-qPCR for SARS-CoV-2 detection.

### 2.7. SARS-CoV-2 RT-qPCR

Routine diagnosis protocol was applied for SARS-CoV-2 detection on NPS and saliva samples by RT-qPCR [15,16,17].

### 2.8. Human RNase P RT-qPCR

RT-qPCR using the *Human RNase P* (HRNP) primers/probe sets were performed as previously described [18] for all saliva samples, in order to ensure the quality of the extraction, also for samples with water addition.

### 2.9. Statistical Analysis

After verifying that values in each group did not assume a Gaussian distribution, the Kruskal-Wallis, Mann-Whitney and Wilcoxon matched-pairs signed-rank tests were computed when appropriate with GraphPad Prism 7.0.0 (GraphPad Software, San Diego, CA, USA). Frequencies were compared by the Chi-square test and confidence intervals reported. All differences were considered significant at *p* < 0.05.

## 3. Results

### 3.1. Clinical Data

A total of 319 samples pairs of NPSs and saliva samples from 145 OP and 51 HW were collected at ten days of interval. Some individuals were lost to follow-up at day ten (*n* = 73), which is frequently encountered in clinical studies. The proportion of missing data at day 10 was around 13.7% (7/51) in the HW group, whereas it reached to 45.5% (66/145) in the OP group. Details about the participants and collection time points were presented in Table 1. No significant differences were noted between age (*p* = 0.932, Kruskal-Wallis test) or gender (*p* = 0.279, df = 3, Pearson’s Chi-square test) among the groups, taking into account collection time point. Nearly two thirds of the OP (*n* = 89, 64.5%) presented symptoms at the enrolment day. The more common symptoms were headache (*n* = 38, 27.5%), tiredness (*n* = 26, 18.8%), cough (*n* = 24, 17.4%), fever (*n* = 21, 15.2%) and myalgia (*n* = 20, 14.5%), corresponding to flu symptoms, frequently described in COVID-19 clinical diagnosis [19,20].

### 3.2. Paired Comparison of SARS-CoV-2 Detection from NPSs and Saliva Samples

Overall, the analysis of the 319 paired sample revealed that the positive rate of SARS-CoV-2 screening by RT-qPCR for NPSs and saliva samples were 39.5% (*n* = 126) and 18.8% (*n* = 60), respectively (Table 2). Among the 126 NPSs samples detected positive for SARS-CoV-2, only 57 saliva samples were confirmed to be infected. If the results from the NPSs were used as reference, saliva samples revealed a low sensitivity of 45.2%. Few false positives were detected in saliva compared to NPSs displaying a specificity of 98.5%. When the kinetic time point collections were taken into account, as expected, the proportions of SARS-CoV-2 positive using NPS specimens decreased between D0 (56.1%; *n* = 110) and D10 (13.0%; *n* = 16). None of the individual detected positive for SARS-CoV-2 by NPSs at D10 were confirmed with saliva specimens (Table 2). 

**Figure 1:**
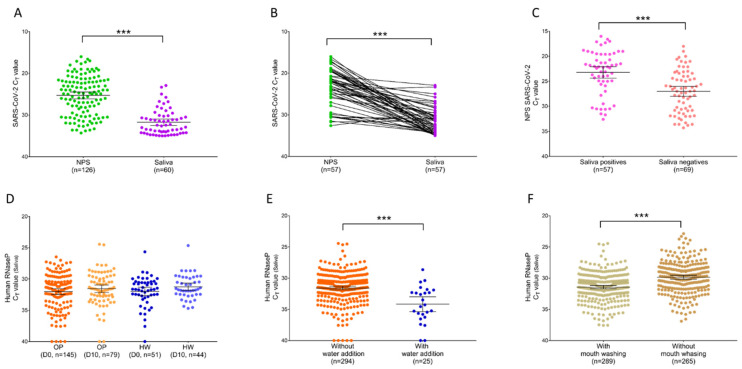
Comparison of Ct values from NPSs and saliva samples. (**A**) Ct values from all SARS-CoV-2 positive NPSs (*n* = 126) and saliva (*n* = 60) samples were compared using a Mann-Whitney U test (*** *p* < 0.0001). (**B**) Paired SARS-CoV-2 positive samples (*n* = 57), represented by the connecting lines, were compared by a Wilcoxon test (*** *p* < 0.0001). (**C**) SARS-CoV-2 Ct values from positive NPS samples found positives (*n* = 57) or negatives (*n* = 69) in saliva specimens were compared by a Mann-Whitney U test (*** *p* < 0.0001). (**D**) Comparison of human RNase P (HRNP) Ct values from saliva samples between outpatients (OP) collected at D0 (*n* = 145), D10 (*n* = 79) and healthcare workers (HW) collected at D0 (*n* = 51), D10 (*n* = 44) (*p >* 0.05, Kruskal-Wallis test). (**E**) Comparison of human RNase P Ct values between saliva samples with (*n* = 25) and without (*n* = 294) water addition (*** *p* < 0.0001, Mann-Whitney U test). (**F**) Comparison of human RNase P Ct values between saliva samples collected with Salivettes without water addition, with (*n* = 265, present work) and without (*n* = 289, previous study [15]) mouth washing before sampling (*** *p* < 0.0001, Mann-Whitney U test). Uniquely significant paired comparisons were indicated. Bars represent the median and 95% CI.

It is interesting to note that all specimens collected on HW (*n* = 95), were found negative for SARS-CoV-2 by RT-qPCR using both specimens at all time points. Comparisons of SARS-CoV-2 results from paired NPSs and saliva specimens of OP are detailed in Table S1. Among the OP enrolled, 75.9% (*n* = 110/145) of the individuals at D0 and 20.3% (*n* = 16/79) of the patients collected ten day later (D10) remained positive for SARS-CoV-2, using NPS specimens for diagnosis. The cycle threshold (Ct) values were significantly higher in saliva than NPS, when all of the samples (*p* < 0.0001, 95% CI (5.195 to 8.145), Mann-Whitney U test, Figure 1A) or paired (*p* < 0.0001, 95% CI (5.87 to 11.20), Wilcoxon test, Figure 1B) samples detected positive for SARS-CoV-2 were considered. The mean of SARS-CoV-2 Ct values increased from 25.3 (95% CI (24.5 to 26.11)) for NPSs to 31.7 (95% CI (30.9 to 32.5)) for saliva samples (Figure 1A), indicating a lower detection of the virus in these last samples. Moreover, the proportion of SARS-CoV-2 detected positive decreased from 56.3% (*n* = 126/224) for NPSs to 26.8% (*n* = 60/224) for saliva specimens (Table S1). Collectively, these results revealed a significantly lower viral load in saliva compared to NPS samples conducting to high proportion of false-negative of SARS-CoV-2 detection, with sensitivity lower than 50% (Table S1). It is likely that mouth washing proposed to participants could induced an alteration of the virus detection in saliva samples.

Interestingly, NPSs SARS-CoV-2 Ct values from OP detected positive also for saliva specimens were found significantly lower than for patients classified positives uniquely by NPSs (*p* < 0.0001, 95% CI (−5.7 to −2.3), Mann-Whitney U test, Figure 1C). Then, the OP positives in saliva for SARS-CoV-2 corresponded to those possessing the higher viral load in NPS specimens, supporting the hypothesis of a dilution effect of mouth washing on virus detection.

### 3.3. Detection of RNA Cellular Control following Mouth Washing

To control whether the mouth washing could be detrimental for RNA detection, the Human RNase P (HRNP) was applied for saliva samples. No comparison could be performed with NPS specimens because they were reserved to SARS-CoV-2 diagnosis and were not available. HRNP was detected in 97.8% (*n* = 312/319) of the saliva samples tested. The Ct values of HRNP were not significantly different between OP and HW groups taking into account the collections time points (*p* = 0.600, Kruskal-Wallis test, Figure 1D), indicating a homogeneity of saliva sampling independently of the groups or time points. Conversely, the addition of ultra-pure water to 25 saliva samples, for which volume retrieved was lower than 150 µL, induced a significant increase of HRNP Ct values compared to those without water addition (*p* < 0.0001, Mann-Whitney U test, Figure 1E), as previously described [15]. These results underlined that impairments of SARS-CoV-2 detection in saliva samples were not attributed to a failing of RNA detection, but rather to an insufficient viral loaded. Water addition induced a significant increase of HRNP Ct values due to dilution of the sample, it is likely that mouth washing could produce a similar phenomenon for RNA from virus. 

To assess the consequence of mouth washing onto RNA detection in saliva samples, a comparison of HRNP Ct between individuals with and without mouth washing before saliva sampling with Salivette^®^ was required. In a recent study, the mean HRNP Ct value obtained in saliva from 265 individuals, collected with Salivette^®^, without mouth washing and without water addition, was 29.85 (95% CI (29.6 to 30.2)) [15]. Here, the mean HRNP Ct value from the 289 individuals, collected with Salivette^®^, with mouth washing and without water addition, was 31.4 (95% CI (31.2 to 31.7)). Although the increase of HNRP Ct values from the mouth washing group was significant (*p* < 0.0001, 95% CI (−1.99 to −1.19), Mann-Whitney U test, Figure 1F), the difference of mean HRNP Ct values between these two groups was modest, about 1.6 Ct. Moreover, the proportion of saliva samples for which HRNP detection failed, were similar in these two groups, 1.5% (*n* = 4/269) and 1.7% (*n* = 5/294) for the previous [15] and for the present study, respectively.

## 4. Discussion

The evidence for the use of saliva as a relevant alternative biological sample to NPSs for SARS-CoV-2 diagnosis has been increasing more and more over time [22]. However, largely, a standardization of saliva collection method and sampling conditions need to be established [23]. Sahajpal et al. [24] pointed out the high sensitivity of saliva use for COVID-19 diagnosis compared to NPSs, even with different saliva collection modes applied. However, a reduction of its performance was noticed in the community evaluated comparatively with the healthcare setting, questioning the great advantage of self-collection of saliva. In contrast to a passive drool into a plastic tube for saliva collection, the commercial devices present the advantages to obtain more consistent sample uniformity [13] and to limit saliva droplets, preventing risk of infection for the health workers [25,26]. Among the various saliva collection systems available, we reported the superiority of Salivette^®^ device for SARS-CoV-2 diagnosis compared to NPS specimens, notably for symptomatic and asymptomatic patients in a recent work [15]. We also observed a miss-paired viral detection in convalescent patients. The weak agreement noticed in follow-up group, was attributed to a viral charge decrease in NPSs [27,28] and saliva samples [29,30], at the first week subsequent to symptoms onset. To confirm performances of saliva sampling with Salivette^®^ compared to NPSs specimens for COVID-19 diagnosis and to assess the level of viral detection concordance in convalescent patients, kinetic paired-sampling were applied.

Here, as numerous individuals ate and drank during the long waiting time, mouth washing with spring water was proposed prior to saliva collection. Mouth washing presented the advantages of limiting the collection of foreign components and homogenizing sampling among participants as it is proposed for biobanking saliva samples [31]. Unfortunately, in contrast to our previous study [15], the proportion of agreement between both specimens was weak for OP (<68%), due to a low sensitivity of saliva samples detecting less than 50% of patients positive by NPSs. The OP who were confirmed COVID-19 positive in saliva samples, corresponded to patients presenting significantly lower SARS-CoV-2 Ct values in NPSs. The dramatic decrease of SARS-CoV-2 detection in positive individuals using saliva specimens was attributed to water mouth washing prior to sample collection. Other saliva factors have been reported to disrupt SARS-CoV-2 detection. The high viscosity of the saliva collected by direct spiting into plastic tube could disrupted the detection of SARS-CoV-2 in the sample [32]. The addition of the homogenization step after saliva collection solved this problem, facilitating saliva pipetting and adequate RNA extraction, improving sensitivity of the assay [32]. Moreover, the heating of saliva samples before the homogenization step allowed to realize RT-PCR tests without the request of RNA extraction for COVID-19 diagnosis [33]. This protocol reduced reagent costs and the sample processing time, which are fundamental requirements for large-scale population screening. As no problem of viscous samples was noticed with Salivette^®^ devices, it will be interesting to assess the performances of SARS-CoV-2 detection in an extraction-free RT-PCR assay.

Numerous studies reported that mouth washing or rinse could reduce and also eliminate SARS-CoV-2 in oral cavity [34,35]. In these studies, buccal rinses were performed with antiseptic mouthwashes, routinely used before dental treatment, to prevent SARS-CoV-2 transmission to dentists. However, to our knowledge, no work assessed the consequence of water mouth washing on SARS-CoV-2 detection in saliva. Mouth washing seems to clean the oral cavity diminishing viral loads, which likely failed to reach the threshold of SARS-CoV-2 molecular detection. Although thirty minutes are currently recommended to wait after eating, drinking or brushing teeth before realizing salivary sampling [23], complementary experiments are required to establish the time needed between mouth washing and saliva collection to avoid virus miss-detection. Others proposed to wait at least 10 min after mouth washing before the process of saliva sampling [36]. The optimization and the determination of appropriate procedure for saliva collection and samples handling until molecular processing need to be improved to implement saliva specimens as relevant alternatives to NPSs for COVID-19 testing [24].

Here, the miss-detection of the coronavirus in saliva samples and the significantly higher SARS-CoV-2 Ct values in saliva compared to NPS samples were not due to an improper saliva sample storing or RNA extraction. Effectively, to control RNA integrity following sampling, sample preservation and extraction, the human cellular control, HRNP, was used as proposed by US CDC [37]. The HRNP was detected in more than 98% of the saliva samples and this rate was comparable using the same collection mode without the prerequisite mouth washing [15]. Moreover, the difference of HRNP Ct values between washed and unwashed mouths prior to saliva collection was modest (about 1.6 Ct). Variations of HRNP Ct values in the same order were obtained for saliva samples tested at successive days corresponding to deviations from replicate experiments [38]. 

The significant decrease of SARS-CoV-2 detection in OP after mouth washing and the remaining detection of the human cellular control (HNRP) in saliva suggest that the virus detection corresponds more to it accumulation in buccal cavity rather than a direct secretion by salivary glands [39]. These data could explain some mitigate results obtained by direct drooling into plastic tubes for COVID-19 diagnosis [40,41]. At the time that saliva tests were carried out to schoolchildren in France, the determination of the performance of saliva collection procedures become imperative.

## 5. Conclusions

The detection of human cellular control, HRNP, in nearly all saliva samples independently of mouth washing and using Salivette^®^ for sampling confirmed that this device appeared as an adequate system for RNA collection in saliva. Conversely, mouth washing decreased viral load of buccal cavity conducting to the impairment of SARS-CoV-2 detection. Viral loads in saliva neo-produced appeared insufficient for molecular detection of SARS-CoV-2. Then, in accordance with others studies that recommend to avoid eating, drinking and tooth brushing at least 30 min before saliva sampling, mouth washing did not allow to rescue individuals who did not respected these instructions. Considering that saliva became a promising source for COVID-19 diagnosis, guidelines concern saliva sampling become mandatory in the near future.

## Figures and Tables

**Table 1:** Characteristics of participants investigated in this study.

	Outpatient Group ^a^		Healthcare Worker Group	
Collection time point ^b^	D0	D10	D0	D10
Participants, n	145	79	51	44
Age (years), median (IQR)	37.3 (23–52)	37.8 (24–51.5)	36.1 (27–45.5)	37.1 (28.0–46.0)
Male, n (%)	71 (49.0%)	42 (53.2%)	22 (43.1%)	20 (45.5%)
Onset of symptoms before D0 test (days), median (IQR)	2.3 (1–3)		/	
Symptoms at presentation, n (%)	94 (64.5%)		0 (0.0%)	
Headache, n (%)	42 (29.0%)		/	
Tiredness, n (%)	27 (18.6%)		/	
Cough, n (%)	25 (17.2%)		/	
Fever, n (%)	25 (17.2%)		/	
Myalgia, n (%)	20 (13.8%)		/	
Breathing difficulties, n (%)	13 (9.0%)		/	
Anosmia/Ageusia, n (%)	9 (6.2%)		/	
Diarrhea, n (%)	8 (5.5%)		/	
Sore throat, n (%)	7 (4.8%)		/	
Others, n (%)	4 (2.8%)		/	

^a^ Tested positively for SARS-CoV-2 by RT-qPCR on NPSs less than five day before enrollment. ^b^ Saliva sampled ten (D10) after the first collection (D0). Abbreviations: IQR, interquartile range; NPS, nasopharyngeal swab; SARS-CoV-2, severe acute respiratory syndrome coronavirus 2.

**Table 2:** Comparison of the RT-qPCR detection of SARS-CoV-2 between NPSs and saliva samples.

Scheme		NPSs						Total
		All Samples (*n* = 319)		Sampled at D0 (*n* = 196)		Sampled at D10 (*n* = 123)		
		Positive	Negative	Positive	Negative	Positive	Negative	
**Saliva**	Positive	57	3	57	1	0	2	60
	Negative	69	190	53	85	16	105	259
	Total	126	193	110	86	16	107	
	Agreement (%)	76.7%		72.5%		85.4%		
	Cohen’s κ ^#^	0.440 (Moderate)		0.475 (Moderate)		NC		
	Sensitivity (%)	45.2%		51.8%		NC		
	Specificity (%)	98.5%		98.8%		98.1%		

^#^ Coefficient of agreement, the agreement level is indicated into brackets, as previously defined [21]. NC: not calculated; NPS, nasopharyngeal swab.

## Supplementary Materials

The following are available online at https://www.mdpi.com/article/10.3390/diagnostics11081509/s1, Table S1: Comparison of the RT-qPCR detection of SARS-CoV-2 between NPSs and saliva samples from the outpatient group.

## Abbreviations

CI: confident interval; COVID-19: Coronavirus Disease 2019; Ct: Cycle threshold; HRNP: Human RNase P; HW: healthcare worker; NC: not calculated; NPS: nasopharyngeal swab; OP: outpatient; PCR: Polymerase Chain Reaction; RT-qPCR: Reverse transcription quantitative real-time PCR; SARS-CoV-2: Severe Acute Respiratory Syndrome Coronavirus 2.
