# Supplementary material for: Mouth Washing Impaired SARS-CoV-2 Detection in Saliva
Source: Diagnostics (Basel). 2021 Aug 22;11(8):1509. doi: 10.3390/diagnostics11081509 (PMC8391436; doi:10.3390/diagnostics11081509)
Supplement: Supplementary file 1 [file diagnostics-11-01509-s001.zip › diagnostics-1304281-supplementary.pdf]

Supplementary Table S1. Comparison of the RT-qPCR detection of SARS-CoV-2 between NPSs and saliva samples from the outpatient group.

Specimens		NPSs						Total
		All samples (n=224)		Sampled at D0 (n=145)		Sampled at D10 (n=79)		
		Positive	Negative	Positive	Negative	Positive	Negative	
Saliva	Positive	57	3	57	1	0	2	60
	Negative	69	95	53	34	16	61	164
	Total	126	98	110	35	16	63	
	Agreement (%)	67.9%		62.8%		77.2%		
	Cohen's κ #	0.392 (Fair)		0.325 (Fair)		NC		
	Sensitivity (%)	45.2%		51.8%		NC		
	Specificity (%)	96.9%		97.1%		96.8%		

#Coefficient of agreement, the agreement level is indicated into brackets, as previously defined [41]. NC: not calculated; NPS, nasopharyngeal swab.
